# Traveling sustainably to and from the EAPS Congress Vienna— a pediatric researcher’s experience

**DOI:** 10.1038/s41390-025-04231-6

**Published:** 2025-07-03

**Authors:** Marie-Louise Herrmann, Mark Hayden, Jasper V. Been, Suzan C. M. Cochius-den Otter

**Affiliations:** 1 Division of Neonatology, Department of Women’s and Children’s medicine, University Hospital Leipzig, Leipzig, Germany; 2 Department of Child and Adolescent Psychiatry, University Medical Center Hamburg-Eppendorf, Hamburg, Germany; 3 Paediatric Cardiac Intensive Care, Great Ormond St Hospital for Children, London, UK; 4 Division of Neonatology, Department of Neonatal and Paediatric Intensive Care, Erasmus MC Sophia Children’s Hospital, University Medical Centre Rotterdam, Rotterdam, The Netherlands; 5 Department of Obstetrics and Gynaecology, Erasmus MC Sophia Children’s Hospital, University Medical Centre Rotterdam, Rotterdam, The Netherlands; 6 Division of Pediatric Intensive Care, Department of Neonatal and Paediatric Intensive Care, Erasmus MC Sophia Children’s Hospital, University Medical Centre Rotterdam, Rotterdam, The Netherlands

## Abstract

- Motivation and call for action for researchers to travel to congresses in a sustainable way.
- Sharing experiences of sustainable traveling regarding the EAPS Congress 2024 Vienna.
- Discussing the pros and cons of sustainable traveling modes for informed decision-making for congress participants.
- Highlighting the impact of congresses on the health care system’s CO_2_ footprint.
- Raising awareness of the need for sustainable congresses.

To the editors, our medical conferences aim to improve pediatric health care. However, because of their environmental impact, they also inflict harm on the planet and—thus—on our patients. Changing our mode of transport can significantly reduce both the CO_2_ footprint and the air pollution caused by pediatric conferences. International flights are responsible for 30–70% of the environmental footprint of medical and scientific activities and are the most harmful mode of transport.^1,2^

We traveled to the EAPS Congress 2024 in Vienna by train and deliberately opted for the most environmentally friendly method of traveling.^2^ By sharing our experience, we hope to inspire others to make a fully informed decision to consider and choose this mode of transport. Our journeys started in Rotterdam (The Netherlands), London (UK), and Trieste (Italy).

Rotterdam–Vienna–Rotterdam: “The outward journey took 14 hours in total. I arrived in the heart of Vienna two hours later due to delays. On the way back, the journey took 13 hours without delays. As the Wi-Fi worked well, I was able to catch up on a lot of work that had been left behind - the autumn colors in the woods were great to look at!”

London–Vienna–London: “If I had to fly, I would not have gone. I caught the Eurostar from London, which departed late, missing my connection in Brussels and getting stuck overnight in Nürnberg- so my travel time outwards was 30 hours and the return 15 hours. While I could justifiably blame Eurostar for their poor performance, a more realistic take is that I left insufficient time to make that first vital connection. I could have caught an earlier train from London and made the trip in one day; lesson learned. Despite the delay, I had a comfortable ride, plenty of time to read, work, and watch the scenery flash by. I was lucky enough to sit next to a seasoned environmental campaigner as we passed by several German coal-burning power plants, something you can’t see in the UK anymore.”

Trieste–Vienna–Hamburg: “On the outward journey, I traveled from Trieste to Vienna by bus. I arrived in Vienna on time after 6 hours. On the way back, I had a direct train connection from Vienna to Hamburg. The journey took 8.45 hours. Due to a timetable change, I had to change trains once, which went without a hitch.”

Climate change is known to be the biggest threat of our time, and CO_2_ emissions from airplanes are a significant contributor to this and to air pollution.^3^ As private individuals, we can reduce our own CO_2_ footprint by choosing our ways of transport. Therefore, congress participants should consider taking the train, bus, or car. With a fully occupied car, the CO_2_ emissions per capita are even lower than when traveling by local train.^4^

Traveling by bus and train also offers several personal advantages: traveling by train can be perfectly combined with a holiday. Attractive stopovers can be made along the way, especially when traveling longer distances to the congress city. Travelers can work on their journeys using the train’s Wi-Fi, using their time well. Chairs in the train are often more comfortable with more leg space compared to traveling by airplane, and on night trains, they can be transformed into beds.

Of course, not all congress participants can do without air transport, for instance, when traveling to a European congress from Australia. Also, time can be a complicating factor. It usually takes longer to travel to a congress by bus or train. For example, a flight from London to Vienna, where EAPS Congress 2024 took place, takes 2.5 h. Traveling to and from the airport and checking in and out also need to be factored in, though. At present, a key barrier is that traveling by plane is often cheaper than traveling by train, largely because of government policies and the absence of a tax on air fuel.

We propose that to make travel by train or bus more mainstream, our employers should also take responsibility and reward sustainable traveling financially and through additional travel time for staff who choose to protect the planet. Night trains can reduce accommodation costs. Even if they are more expensive, they can be cost-effective. Congress planners should promote sustainable travel and avoid meetings in cities with poor train connections. Hybrid congresses with the option of online participation should be a mandatory requirement.^5^ Alternating in-person and online conferences and decentralizing conferences to multiple venues in different countries would be sustainable alternatives.^1^ On a higher level, policy-makers nationwide must expand rail connectivity to enable sustainable travel across national borders.

If individuals in more hospitals and research labs opted for a mode of sustainable transport, they would serve as role models and motivate others to travel sustainably. You can even make a game of it. For instance, a competition was started between departments at Erasmus MC in Rotterdam. Annually, the department with the most travelers who have taken the train to conferences wins a prize.

Sustainable travel is only part of the solution. It does not absolve conference organizers from addressing other harms like plastic waste, single-use coffee cups, bottled water, carbon-intensive foods such as meat and cheese, and accommodation.^5^ Congress planners must pursue a sustainable concept and report travel mode data for participants and estimated carbon emissions. And we should also choose wisely: does the carbon footprint of an overseas conference really weigh up to the impact of your attendance? Or can you participate digitally just as well? At least, does the Congress provide a sustainable concept?

As medical professionals, especially as pediatricians and pediatric researchers, we are ethically bound to foster a healthy future for our young patients in a healthy environment. Let’s start by taking the train!
